# Live Cell Monitoring of Separase Activity, a Key Enzymatic Reaction for Chromosome Segregation, with Chimeric FRET-Based Molecular Sensor upon Cell Cycle Progression

**DOI:** 10.3390/bios14040192

**Published:** 2024-04-15

**Authors:** Md. Shazadur Rahman, Yutaka Shindo, Kotaro Oka, Wataru Ikeda, Miho Suzuki

**Affiliations:** 1 Graduate School of Science and Engineering, Saitama University, 255 Shimo-Okubo, Sakura-ku, Saitama 338-8570, Japan; rahman.m.s.902@ms.saitama-u.ac.jp (M.S.R.); w.ikeda.927@ms.saitama-u.ac.jp (W.I.); 2 Department of Agricultural Chemistry, Hajee Mohammad Danesh Science and Technology University, Dinajpur 5200, Bangladesh; 3 Department of Bioscience and informatics, Faculty of Science and Technology, Keio University, Yokohama 223-0061, Japan; shindo@z5.keio.jp (Y.S.); oka@bio.keio.ac.jp (K.O.); 4 School of Frontier Engineering, Kitasato University, 1-15-1 Kitasato, Minami-ku, Sagamihara 252-0373, Japan

**Keywords:** separase, cohesion, securin, CDK-1, cyclin B1, cell cycle, chromosome segregation, FRET, live cell sensing

## Abstract

Separase is a key cysteine protease in the separation of sister chromatids through the digestion of the cohesin ring that inhibits chromosome segregation as a trigger of the metaphase–anaphase transition in eukaryotes. Its activity is highly regulated by binding with securin and cyclinB-CDK1 complex. These bindings prevent the proteolytic activity of separase until the onset of anaphase. Chromosome missegregation and aneuploidy are frequently observed in malignancies. However, there are some difficulties in biochemical examinations due to the instability of separase in vitro and the fact that few spatiotemporal resolution approaches exist for monitoring live separase activity throughout mitotic processes. Here, we have developed FRET-based molecular sensors, including GFP variants, with separase-cleavable sequences as donors and covalently attached fluorescent dyes as acceptor molecules. These are applicable to conventional live cell imaging and flow cytometric analysis because of efficient live cell uptake. We investigated the performance of equivalent molecular sensors, either localized or not localized inside the nucleus under cell cycle control, using flow cytometry. Synchronized cell cycle progression rendered significant separase activity detections in both molecular sensors. We obtained consistent outcomes with localized molecular sensor introduction and cell cycle control by fluorescent microscopic observations. We thus established live cell separase activity monitoring systems that can be used specifically or statistically, which could lead to the elucidation of separase properties in detail.

## 1. Introduction

Separase is a large eukaryotic endopeptidase (140-250 kD) belonging to the cysteine protease family [1] which performs critical functions in the maintenance of genetic homeostasis. Cells need to maintain genome stability during cell division to preserve and transmit their hereditary material intact to the next generation [2]. The cell cycle is a dynamic biological process that causes cells to divide into two daughter cells, and the most dramatic cellular phenomenon involved in the cell cycle is the irreversible separation of sister chromatids, which happens during the mitotic phase, specifically at the metaphase–anaphase transition in eukaryotes. These precisely controlled processes are triggered by separase activation which is tightly regulated by its inhibitory chaperone, securin [3,4,5]. Securin stabilizes separase by co-translational binding to assist its correct folding [6,7,8]. The CDK-1 in vertebrates phosphorylates to connect phosphorylated separase and cyclin B1 to form the other controlling complex for separase activity [9,10,11].

Generally, separase has vital roles in many cellular functions, besides chromosome segregation during mitosis and meiosis [1,3,12], DNA damage repair [13,14,15], centrosome disengagement and duplication [16,17], and spindle stabilization and elongation [18,19]. The proteins astrin and Aki1 function as inhibitors of centrosomal separase [20] and these combinations engage in proper spindle function in mitosis [21]. Separase disintegrates the cohesion between sister chromatids by cleaving one of the subunits Scc1/Rad21/Mcd1 from the cohesin ring for chromosomal segregation [22]. Cohesin is the “glue” that binds the mother and daughter centrioles, and cleavage of this pool of cohesin by separase encourages centriole disengagement, according to various biochemical investigations [23]. Interestingly, separase cleaves both the cohesin subunits Scc1/Rad21/Mcd1 and kendrin/pericentrin B at the centrosome [24]. But separase is well known to indicate the minimal consensus motif as ExxR at cleavage sites [1,4,22]. Separase activation is elicited by destruction of securin [25,26,27] and cyclin B1 [9] by the proteasome degradation system after ubiquitination with anaphase-promoting complex/cyclosome (APC/C) [28,29]. Additional mechanisms for the separase activation process have been reported, such as that auto-cleavage of separase is needed to promote mitosis, but not to enhance protease activity [18,30,31,32]. These activation profiles are directed through phosphorylation states of separase including specific protein phosphatase 2A [33,34,35]. The molecular mechanisms underlying separase behaviors have been revealed by recent structural studies [36,37,38,39].

**Figure 1:**
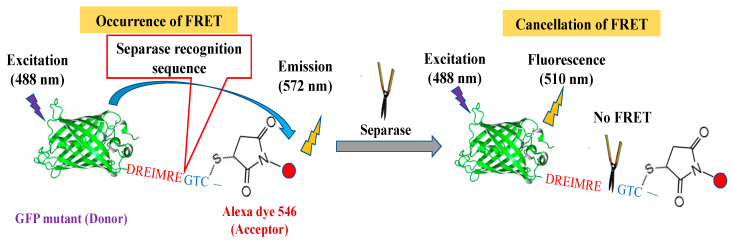
Sensing mechanism of FRET-based separase molecular sensors.

However, there are still questions remaining about the activities and regulation of separase activities which can be clarified with biochemical approaches. It is partially due to problems in defective expression systems that it is difficult to obtain active separase unassisted by a chaperone protein [7,40]. It is possible for anti-cancer medications to establish an index that will normalize excessive or insufficient separase activities. Therefore, in this investigation, we try to detect separase activity in mammalian live cells through FRET-based sensing by changing intramolecular FRET efficiencies upon enzymatic reactions. We have designed a chimeric FRET-based molecular sensor that utilizes a green fluorescent protein (GFP) mutant (donor molecule) with separase-cleavable sequences followed by unique cysteine for chemical modification with a fluorescent organic dye (acceptor molecule) as a simple conjugate (Figure 1) [41]. We could easily replace the donor fluorescent protein, recognition sequences, and dye species to optimize the assay system [42]. Furthermore, the molecular sensor can efficiently be delivered into live cells via endocytotic pathways. Here, we have attempted to localize our molecular sensor for monitoring organelle-specific events such as separase activations. We introduced microscopic observation to confirm our aim, and flow cytometric analysis to check the variety in the activation extent for separase during cell cycle progression, using a molecular sensor localized inside the nucleus as well as a non-localized one. We succeeding in detecting separase activations upon pushing the cell cycle forward in every case equally, demonstrating that our robust molecular sensor functioned beyond existing approaches. The achievable flow cytometric separase assay would render the range of intracellular variation in separase activity or its alteration upon anti-cancer drug treatment in combination with other fluorescent markers or microscopically detailed observations. Very impressively, the versatile molecular sensor contributory systems we developed here can potentially not only monitor the dynamics of separase in situ, but also can assess the real-time therapeutic efficacy of cancer treatment.

## 2. Materials and Methods

### 2.1. Plasmid Construction for Separase Activity Detection with or without Nuclear Localization Sequence (NLS)

Inverse polymerase chain reaction (iPCR) [43] was used to construct a plasmid containing the reporter genes for GFP and to detect separase activity. This plasmid was based on the pUV5casS52tag for caspase-3 activity sensing [44,45] and the first manipulation was caried out to replace DEVD-coded regions with DREIMRE around the C-terminal and to adjust linker sequences for the accessibility of the target enzyme referring to the molecular sensor for caspase-9 [add1] because of its structural similarity with separase, to create pUV5Sep. The second manipulation included the insertion of GPKKKRKV (NLS) [46,47] around the N-terminal to obtain pUV5SepNLS. The genetic manipulations above were certified by the usual method for gene sequencing. Coded amino acid sequences for the three GFP variants mentioned above are shown in Figure S1.

### 2.2. Chimeric FRET-Based Molecular Sensor Preparation

Two variant green fluorescent proteins (GFP) were isolated initially. The two plasmids described above (pUV5Sep and pUV5SepNLS) were transfected into *Escherichia coli* BL21 cells (DE3). The transfected bacteria were cultured in Luria Bertani (LB) medium containing 75 µg/mL of ampicillin; the expression of the GFP derivatives was induced by isopropyl-β-D-thiogalactopyranoside (IPTG). The bacteria were harvested and lysed with a sufficient amount of bacterial protein extraction reagent, B-PER II (Thermo Scientific Pierce, Rockford, IL, USA). Each target protein was isolated from the lysate by centrifugation for separation from cell debris and purified using Ni2+-NTA (nitrilotriacetic acid) affinity chromatography through (Histidine)6 tag sequence fused with the GFP derivative. The remaining reagents were removed with a gel filtration column and recovered proteins were then reconstituted in phosphate-buffered saline (PBS) separately. Isolated fluorescent proteins (FP) were chemically modified with fluorescent dyes, namely Alexa Fluor-546 (Life Technologies, Carlsbad, CA, USA) (Thermo Scientific Pierce). Then, 10 µM of GFP dissolved in PBS was reduced with 1mM of dithiothreitol (DTT). Excess DTT was removed by gel filtration equilibrated with phosphate-buffered saline (PBS) (NICK column, GE Healthcare, Buckinghamshire, UK), and an aliquot of the eluate was immediately incubated with the corresponding fluorescent dye at the molar ratio of 1:10 at 37 °C for 4 h which was then allowed to stand at 4 °C for 6 h. Ultrafiltration (EMD, Millipore, Billerica, MA, USA) was used to remove unreacted dye and concentrate the solution to an adequate volume for use in future studies. The FRET efficiencies were assessed to check emission profiles excited at 488 nm by fluorescence spectroscopy using a Shimadzu RF-5300PC spectrophotometer (Shimadzu, Kyoto, Japan).

### 2.3. Evaluations for Separase Activity with Chimeric FRET-Based Molecular Sensor

We have already demonstrated that a requisite minimum formula to approximate FRET efficiency, fluorescence intensity of emission maxima for the donor molecule, and fluorescence intensity of emission maxima for the acceptor molecule could be applied to quantify target enzyme activities for most constructs of GFP variants and Alexa Fluor-532, 546, 555, 594, due to the high FRET efficiencies of the chimeric molecular sensors followed by calculations for increasing the ratio of FRET efficiencies [45]. GFP variants for caspase-3 and caspase-9 detections attached with Alexa Fluoro-546 were subjected to further evaluations as molecular sensors on the points by measuring fluorescence lifetime changes of donor molecules upon enzymatic reactions [48]. As estimated, the Förster radius for those constructs supported the high FRET efficiencies. Some extracted estimations are shown in Supplementary Table S1.

### 2.4. Introduction of Chimeric FRET-Based Molecular Sensor into Cell-Cycle-Controlled HeLa Cells

The chimeric FRET-based molecular sensor was confirmed to be introduced into cells through straightforward methods by the incubation of cells with culture media including molecular sensors. We have already demonstrated efficient uptakes of many combinations of GFP variants and Alexa dyes into several cell types [45]. As endocytic pathways could be involved as mechanisms for the uptake due to partial inhibitions with some endocytic inhibitors and no effects at all from serum abundance or deprivation [unpublished data], we have thus introduced the chimeric FRET-based molecular sensor according to an approach based on cell cycle control. Subcultured HeLa cells were seeded into a culture dish filled with Dulbecco’s Modified Eagle Medium (DMEM) supplied with 10% fetal bovine serum (FBS) at 37 °C under an atmosphere containing 5% CO_2_. After confirmations of proper growth, the culture medium was replaced with DMEM without FBS to suppress cell cycle progression and incubated for 24 h at 37 °C under an atmosphere containing 5% CO_2_. Following this, the culture medium was replaced with DMEM without FBS containing 5 μM of corresponding FRET-based molecular sensor for 12 h incubation at 37 °C under an atmosphere containing 5% CO_2_. The residual FRET-based molecular sensor was removed by washing the cells with culture medium without FBS and subjected to further experiments. 

### 2.5. Flow Cytometry

The HeLa cells introduced by the FRET-based molecular sensor, along with further experimentally processed cells, were rinsed with 250 µL of 0.2 mM EDTA. The chelating solution was removed to stock and the remaining cells were treated with 250 µL of Accutase and Accumax cell detachment solutions (Innovative Cell technologies, San Diego, CA, USA) for 5 min at 37 °C. The detached cell mixtures were recovered and combined with the stocked solutions. The remaining cells were completely detached with 250 µL of 0.25% trypsin solution. The resulting cell mixtures were added to all the combined solutions that had been so far produced at this point in the process. The culture plate was washed with 250 µL of PBS to be transferred to all the recovered solutions described above. The resulting solutions were filtered before being subjected to flow cytometry analysis using a SONY FACS machine (LE-SH800 SONY Co., Ltd. Tokyo, Japan). The fluorescence intensity and other optical data of the harvested cell population (30,000 cells) were monitored by excitation using a 488-nm semiconductor laser and adequate filter sets were selected to split donor (GFP variants) and acceptor (Alexa Fluor-546) emissions. FlowJo software (version10.7.2) (Becton, Dickinson and Company, San Jose, CA, USA) was used to process the collected data.

### 2.6. Verification of Cell Cycle Contorol by Serum Starvation through DNA Amount Estimation

Harvested subconfluent cells were centrifuged at 15,000 rpm and resuspended in 1 mL of DMEM supplied with 10% FBS and 10% DMSO. The suspension was frozen for one hour at −20 °C. The resulting materials were thawed and centrifuged at 15,000 rpm at 4 °C to remove supernatant. Collected cells were resuspended to adjust recommended cell density as 1 × 10^6^ cells per milliliter in 1 ml of ice-cold Hoechst staining buffer and incubated at 4 °C for 15 min in the dark. Ethidium bromide was added to 1 g/mL solution and allowed to stand for 15 min at 4 °C in the dark. All the solutions were filtrated and the DNA contents of the recovered cell solutions were determined using flow cytometric analysis as described above. Data analysis was performed with Flow Jo software correspondingly. 

### 2.7. Separase Activations through Cell Cycle Progression

The HeLa cells introduced by the FRET-based molecular sensor, as described in Section 2.3, were divided into two populations. One population was harvested to be applied to flow cytometric analysis directly. The other populations were continuously exposed to DMEM supplied with 10% FBS at 37 °C under an atmosphere containing 5% CO_2_ for 24 h. Processed cells were then collected and analyzed by flow cytometry, as mentioned above. 

### 2.8. Fluorescence Microscope Observation of Bioprobe Localization inside Cells

The HeLa cells introduced by the FRET-based molecular sensor, described in Section 2.3, were divided into two populations. One population was observed immediately before the cell cycle restarted and the other was observed after 24 h of exposure to DMEM supplied with 10% FBS, as the population after cell cycle progression. Fluorescence imaging was performed at room temperature using an FV-1000 confocal microscope (Olympus, Tokyo, Japan) with a ×60 oil immersion objective lens. The FRET-based molecular sensor was excited at 488 nm from an Ar laser through a dichroic mirror (DM405/488/559). Fluorescence was separated at 560 nm by a dichroic mirror and detected by two photomultipliers through suitable band path filters: 500–545 nm for GFP and 570–670 nm for Alexa 546.

### 2.9. Imaging Analysis

The acquired images were analyzed with FV10-ASW software (Olympus). Regions of interest (ROIs) were placed on the nucleus region on the sensor-introduced cells, and the average fluorescence intensity of each ROI was calculated. After the average fluorescence intensity of the ROI at the region without cells was subtracted as background, the fluorescence ratio (GFP/Alexa 546) was calculated.

### 2.10. General Image Acquisitions for FRET-Based Molecular Sensor Localizations in HeLa Cells

Approximate examination of molecular sensor localization was carried out at room temperature using an ECLIPSE TE 2000U (Nikon Solutions Co., Ltd., Tokyo, Japan) with a ×40 water immersion objective lens. The FRET-based molecular sensor was excited with an ultra-high pressure mercury lamp through an adequate combination of a dichroic mirror and filter to detect GFP and Alexa 546 emissions and show bright images of each. The acquired images were processed with ImageJ supported by NIH.

### 2.11. Western Blot Analysis

Expression profiles of separase for cell populations in cell cycle arrest or its continuation were examined by Western blotting. HeLa cells cultured with DMEM supplied with or without 10% FBS for 24 h were collected individually by centrifugation at 15,000 rpm following trypsinization. The recovered cells were rinsed with aliquots of PBS by centrifugation by the same method and lysed using M-PER™, Mammalian Protein Extraction Reagent (Thermo Fisher Scientific Inc., Waltham, MA, USA), according to the standard procedures as instructed by the supplier. Protein concentrations for recovered extracts were determined by spectrophotometer at 280 nm wavelength absorption. Denatured samples were applied to SDSPAGE and transferred to the PVDF membrane to carry out Western blotting using anti-Separase rabbit polyclonal antibody (abcam plc. Cambridge, UK) as the primary antibody, followed by detection of the 233 kDa full-length separase with Goat Anti-Rabbit IgG HL (Alexa Fluor-680) as the secondary antibody. Band assignment for the full-length and cleaved separase was carried out using prestained protein standard (Bio-Rad laboratories, Inc., Tokyo, Japan). Protein extraction of cell lysates was verified for adjustment by glyceraldehyde 3-phosphate dehydrogenase (GAPDH) detection with GAPDH rabbit polyclonal antibody (Proteintech Group Inc. Tokyo, Japan). Band images were taken by fluorescent imager (Typhoon FLA 9500, GE Healthcare Japan, Tokyo, Japan).

### 2.12. Separase Activity Estimation Included in Cell-Cycle-Progressed Cell Lysate Preparation with FRET-Based Molecular Sensors

We examined the separase activity derived from the cell-cycle-progressed cell lysate preparations mentioned in Section 2.11 using two types of chimeric FRET-based molecular sensors which were NLS-based and WNLS-based. We developed a modified method that we utilized to validate the FRET-based molecular sensors in vitro with a commercially available enzyme. Briefly, 2 μM of each FRET-based molecular sensor was incubated with the obtained cell lysate for 1 h. As a control experiment, we incubated the molecular sensors with aliquots of M-PER™. After 1 h of incubation, we assessed the emission profiles excited at 488 nm by fluorescence spectroscopy.

## 3. Results and Discussion

### 3.1. FRET-Based Molecular Sensor Preparations and Their Fluorescence Properties

We initially produced molecular sensors to target separase protease. We hypothesized that it would be straightforward to utilize a Fluorescence Resonance Energy Transfer (FRET) system as the sensing mechanism for proteolysis. We established chimeric FRET-based molecular sensors for varied cysteine proteases as caspase-1, 3, 9, and 14 [47,48,49]. Though we have already demonstrated a broad range of Alexa dyes (Alexa 532, 546, 555, 568, and 594), these combined with GFP variants could perform emission spectrum changes upon proteolytic monitoring; there were preferable dyes for individual cysteine proteases. The structural basis for separase belonging to the cysteine protease family confirmed it to be highly homologous to caspase-9 [36], and thus we introduced Alexa 546 for our new targeted FRET-based molecular sensors because it is the optimal dye for caspase-9.

**Figure 2:**
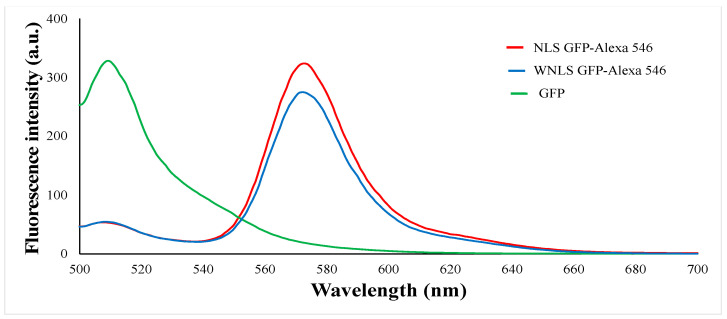
Emission patterns of molecular sensors excited at 488 nm.

Next, we examined fluorescence emission patterns obtained upon the attachment of the dyes to two GFP variants with or without Nuclear Localization Signal (NLS), namely NLS-based and WNLS-based (without NLS), to evaluate molecular sensors regarding FRET-based sensing by FRET efficiencies of the corresponding molecular sensors (amino acid sequences are shown in Figure S1). For each combination, significant decreases in donor fluorescence intensities and appearances of acceptor emissions were observed, as shown in Figure 2. The emission ratios were estimated as approximate FRET efficiency = donor emission intensity maximum/acceptor emission intensity maximum. The calculated FRET efficiencies of the NLS-based and WNLS-based variants were 0.164 and 0.200, respectively. In the case of commercially available protease, we can assess sensing performances as emission spectrum changes with protease treatments to obtain exchanges for FRET efficiencies; however, inaccessibility for isolated separase due to unstable behavior by dissociation from endogenous chaperone protein might prevent such operations. Therefore, tracking separase activities through live cell imaging or flow cytometric analysis could be effective for that purpose. Additionally, those high FRET efficiencies might promise to quantify separase activities in a single cell through uncomplicated data processing as emission maxima of donor molecule/emission maxima of acceptor molecule, as explained in the Section 2.

### 3.2. Introduction of Chimeric FRET-Based Molecular Sensors into Cell-Cycle-Controlled Hela Cells and Flow Cytometry 

**Figure 3:**
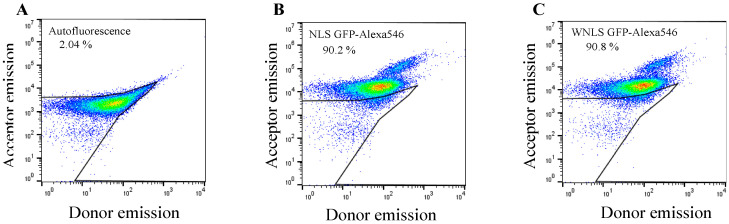
Flow cytometric confirmation of molecular sensors for separase-sensing uptake into HeLa cells by dot plot analysis.

After successful preparation of molecular sensors, we next assessed the molecular sensors’ uptake efficiencies into HeLa cells using flow cytometry. The cell sets were subjected to donor and acceptor emission dot-plot analysis for untreated cells, and treated cells in two groups: NLS GFP-Alexa 546 and WNLS GFP-Alexa 546. By gating the untreated cell populations for auto fluorescent estimation, we confirmed that more than 90% of cell populations could uptake a significant amount of the fluorescent proteins by applying a 5 μM concentration of molecular sensors. Moreover, there were no big differences between the molecular sensors analyses of the two GFP variants, as shown in Figure 3A–C.

### 3.3. Checking Cell Cycle Control/Synchronization by DNA Quantification 

**Figure 4:**
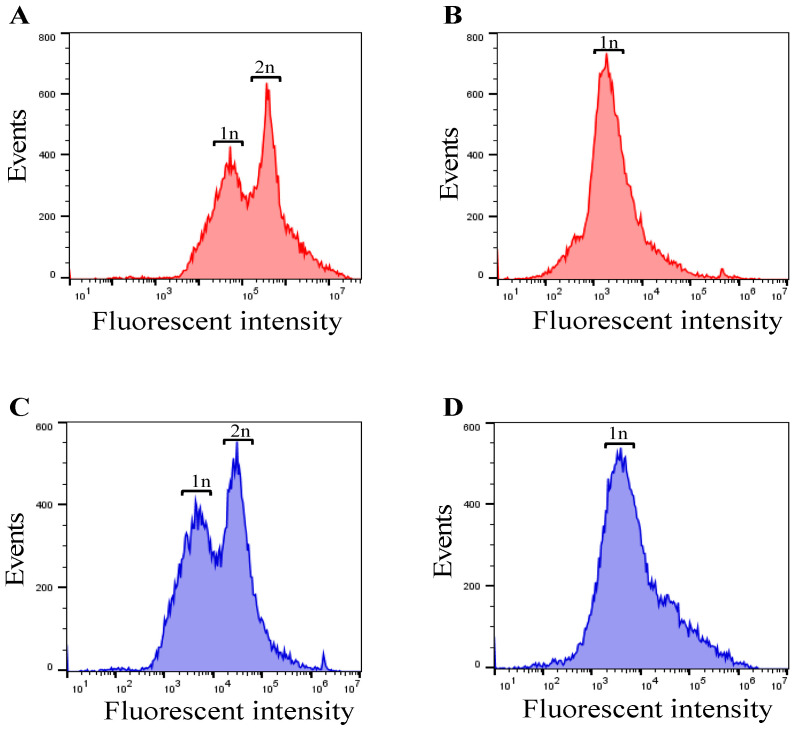
DNA amount in FBS-supplied (100% FBS) and FBS-starved (0% FBS) HeLa cells stained with ethidium bromide (**A**,**B**) and propidium iodide (**C**,**D**).

As separase activities in cells and proliferation rates of the cell population should correlate with the amount of DNA in the cells, we examined the influences of cell cycle synchronization by serum starvation [50] on the amount of DNA contained in the cells. HeLa cells which had undergone 48 h serum deprivation were stained with ethidium bromide and propidium iodide to compare with a non-serum-starved HeLa cell population and the DNA content (Figure 4) was measured using a flow cytometer (SONY FACS machine). In the case of the serum starvation condition, only haploid cells were found, whereas in the cell population with the serum supplement condition both haploid and diploid cells were observed. We thus proved that cell cycle arrest emerged as cells with haploid DNA content. Accordingly, we could collect separase-activated cells through re-feeding with serum for cells.

### 3.4. Separase Activations through Cell Cycle Progression

Combined with the results of our investigations into molecular sensor uptake efficiency and cell cycle synchronization by serum supplement conditions, we next tried to monitor separase activities with molecular sensors localized inside the nucleus or dispersed in the whole cell upon separase-activated cell collection. First, we observed localizations for two types of molecular sensors inside HeLa cells through the microscopic observations mentioned in Section 2.7. As shown in Figure S2, we obtained different distribution patterns for each molecular sensor. As for the non-localized type of molecular sensor (WNLS-based), it seemed to stay at the endosome around the nucleus to disperse into the cytosol. On the other hand, the nucleus-localized type of molecular sensor (NLS-based) accumulated inside the nucleus. 

**Figure 5:**
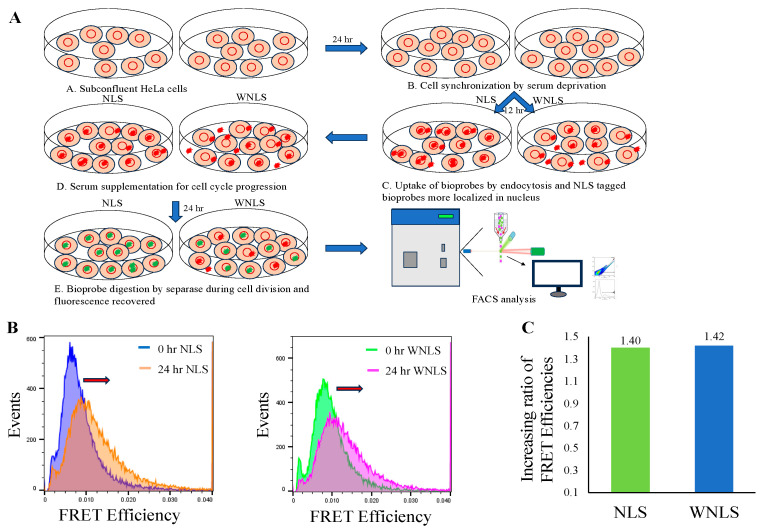
Separase activity monitoring through cell cycle progression. (**A**) Cell cycle control experimental flow was schematically drawn. (**B**) FRET efficiencies (after 0 h and 24 h incubation) were approximately estimated using median values from histograms for FRET efficiencies. (**C**) Increasing ratios for FRET efficiencies of 0 h and 24 h were calculated for NLS- and WNLS-based molecular-sensor-introduced cell populations.

After confirmation of different localizations in cells for NLS- and WNLS-based molecular sensors, we measured separase activities upon cell cycle control, as shown in Figure 5A. We then checked distributions of FRET efficiencies for molecular sensors inside HeLa cells as histograms derived from populations using dot plot analysis (Figure 5B). Both cell populations demonstrated FRET efficiency increases after 24 h of re-feeding with serum. We calculated increasing ratios for 0 h to 24 h (FRET efficiencies at 24 h/FRET efficiencies at 0 h), and the calculated ratio for the NLS-based molecular sensor (1.40) was nearly the same as that for the WNLS-based one (1.42) (Figure 5C). This indicates that these molecular sensors would be adequately sensitive to detect separase activity inside cells and a sufficient amount of non-localized molecular sensor might spread to inside the nucleus.

### 3.5. Imaging Analysis

**Figure 6:**
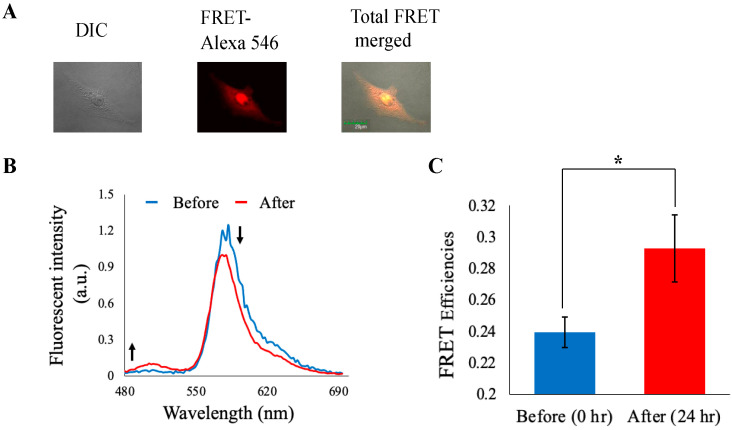
Separase activity monitoring through cell cycle progression. (**A**) Fluorescence microscopic observation of molecular sensor localization inside cells. (**B**) The emission profiles in HeLa cells after 0 h and 24 h incubation in culture medium containing serum. Fluorescence intensity was normalized at isosbestic point. (**C**) FRET efficiency exchanges before and after cell cycle progression (Before: *n* = 55 cells from three different experiments, After: 34 cells from three different experiments). Error bars: S.E.M. *: *p* < 0.05 in Student’s *t*-test.

We then attempted to validate our NLS-based molecular sensor function using microscopic observations in detail upon corresponding cell cycle control. As shown in Figure 6A, it was demonstrated that the NLS-based molecular sensor almost reached the inside of the nucleus normally. Some molecular sensor uptake cells changed their shape to spherical but indicated equivalent localization patterns, as shown in Figure S3. Though the introduction process for the molecular sensor into cells was common with flow cytometric analysis, sensitivities to fluorescence intensity were low according to microscopic observations. In previous studies, detection limits for live cell imaging were estimated around 2 × 10^2^ for donor molecule emission and 5 × 10^4^ for acceptor molecule emission, referring to Figure 3. Based on this estimation, an approximate average of 1% of cell populations were predicted to exceed those ranges. The percentage meeting these criteria did not render any difficulties in finding proper cells for fluorescence microscopic observations. Representative emission profiles of them are shown in Figure 6B. A small but steady increase in GFP fluorescence was observed, indicating disappearance of FRET between GFP and Alexa 546 upon digestion of the inserted separase recognition sequence. We also calculated and compared the average FRET efficiencies for cells processed for 0 h and 24 h (Figure 6C). FRET efficiency (GFP/Alexa 546) increased after cell cycle progression. These observations almost fitted with our flow cytometric analysis, i.e., our chimeric FRET-based molecular sensor could appropriately detect separase activities upon cell cycle progression.

### 3.6. Western Blot Analysis for Separase Expression 

**Figure 7:**
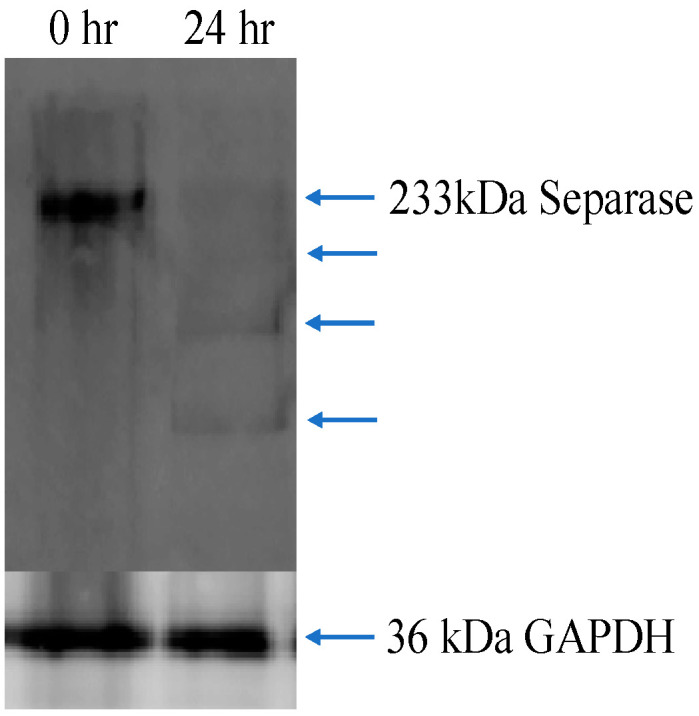
Separase expression profiles of cell cycle synchronized 0 h and 24 h. Blue arrow heads point to assigned bands as intact and autocleavage of separase. Comparable patterns were obtained from two more lysate preparations.

We analyzed separase occurrences for cells under cell cycle control as 0 h and 24 h processing by Western blotting. As shown in Figure 7, separase occurrences in both cell populations were found. However, a more clear presence of separase was shown in the cell-lysate-derived sample that was processed for 0 h. On the other hand, the 24 h treatment indicated not only a faint presence of separase but also several digested bands. These findings might be consistent with separase occurrences bound with the chaperone protein securin and CDK1-cyclin B1 complex to inhibit their activity before cell cycle progression and separase activation through release from those inhibitors followed by autocleavage [51,52]. It was reported in previous studies on human-originated separase that some autocleavable sites rendered analogous digested patterns consisting of three main bands, as a faint band at less than 150 kD, a clear band around 100 kD, and another clear band around 70 kD, during cell cycle progressions. Our band identification based on molecular markers generally matched the findings mentioned above.

### 3.7. Separase Activity Estimation Derived from Cell-Cycle-Progressed Cell Lysate

**Figure 8:**
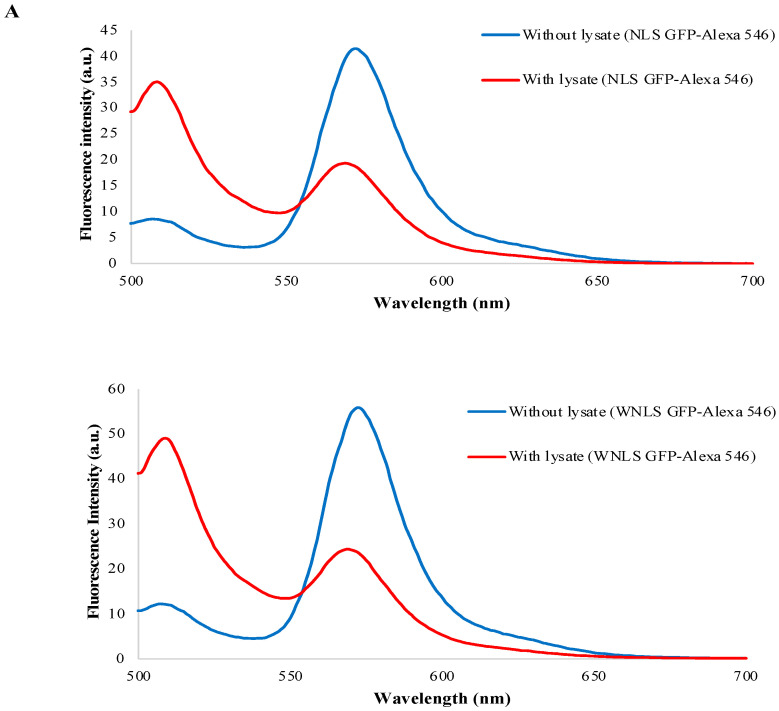

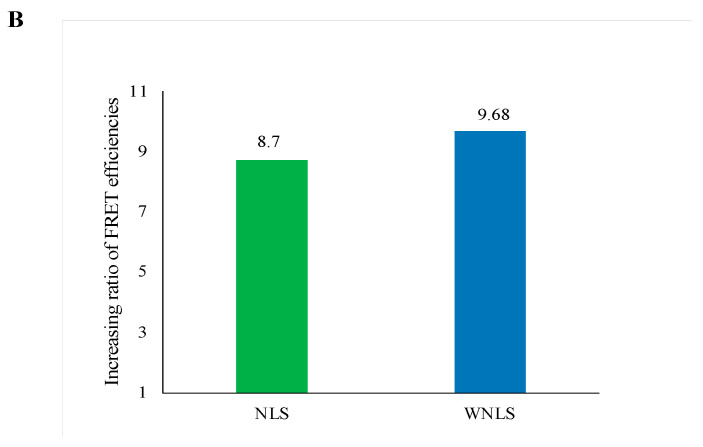
Separase activity validation contained in lysate preparations. (**A**) Emission pattern changes of molecular sensors through lysate treatments. (**B**) Increasing ratio of FRET efficiencies through lysate treatments.

As we could obtain activated separase fractions from cell-cycle-progressed lysate preparations mentioned above, we investigated the separase activities they contained using two types of FRET-based molecular sensors. As shown in Figure 8A, we detected drastic exchanges of FRET patterns in both molecular sensors. The calculated increasing ratios of FRET efficiency were 8.70 for the NLS-based variant and 9.68 for the WNLS-based variant (Figure 8B). We tried to perform corresponding experiments using 1/5 and 1/50 diluted lysate preparations and achieved consistent as dose-dependent results. A slightly more sensitive performance of separase activity detection in the lysate preparations was repeatedly found in every WNLS case. These results implied that constructs for the WNLS-based type could be accessible for separase with high molecular weight and were consistent with the results shown in Figure 5C. 

## 4. Conclusions

We have established live cell monitoring systems for separase activity through microscopic observation and flow cytometric analysis that could allow quantitative assay for separase activation profiles during cell cycle with spatiotemporal resolution and statistic considerations owing to consistent outcomes from both approaches. Our monitoring systems could easily be applied to other cell types [42]. This could render new insight for separase behaviors in live cells under cell cycle arrest or progress conditions in detail. Furthermore, our simple strategy to fabricate chimeric FRET-based molecular sensors for live cell proteolysis monitoring enables us to improve sensitivities, replace target proteases, alter localization inside cells, and change fluorescent properties by protein engineering and introductions of other combinations for fluorescent proteins and dyes. It might expand sensing targets simultaneously. Combinatorial use of molecular sensors already demonstrated for caspase-9 and -3 might straightforwardly be substituted by the sensor in this work, which would give us advanced comprehension for separase involved in programmed cell death [53]. In this context, we are confident that our assay will be applicable as a clinical diagnostic and prognostic tool to monitor separase proteolytic activity combined with other cellular events in human malignancies, and as a therapeutic mechanism in future studies.

## Data Availability

The original data are available upon request from the corresponding author.

## Supplementary Materials

The following supporting information can be downloaded at: https://www.mdpi.com/article/10.3390/bios14040192/s1, Figure S1: Amino acid sequences for molecular sensor of separase; Figure S2: General fluorescence microscopic observation to compare two types of molecular sensor localizations in cells. It appeared that not localized type of molecular sensor (WNLS based) stays at endosome surrounding the nucleus to disperse into the cytosol. On the other hand, nucleus localized type of molecular sensor (NLS based) appeared to be accumulated inside nucleus; Figure S3: Identification of different cell states upon NLS based molecular sensor uptake using fluorescence microscopic observations in detail. Although forms of introduced cells were varied as a spherical shape or spread one, they still exhibited identical localization patterns for molecular sensors; Table S1: Estimated Forster Radius.
