# Supplementary material for: Live Cell Monitoring of Separase Activity, a Key Enzymatic Reaction for Chromosome Segregation, with Chimeric FRET-Based Molecular Sensor upon Cell Cycle Progression
Source: Biosensors (Basel). 2024 Apr 15;14(4):192. doi: 10.3390/bios14040192 (PMC11048197; doi:10.3390/bios14040192)
Supplement: Supplementary file 1 [file biosensors-14-00192-s001.zip › biosensors-2896337-supplementary.pdf]

Supplementary Information

Live cell monitoring of separase activity, a key enzymatic reaction for chromosome segregation, with chimeric FRET-based molecular sensor upon cell cycle progression

Md. Shazadur Rahman [1,2], Yutaka Shindo[3], Kotaro Oka[3,4], Wataru Ikeda[1] and Miho Suzuki[1*]

[1] Graduate School of Science and Engineering, Saitama University, 255 Shimo-Okubo, Sakura-ku, Saitama, 338-8570, Japan

[2] Department of Agricultural Chemistry, Hajee Mohammad Danesh Science and Technology University, Dinajpur-5200, Bangladesh

[3] Department of Bioscience and Informatics, Faculty of Science and Technology, Keio University, Kanagawa, 223-0061, Japan

[4] School of Frontier Engineering, Kitasato University, 1-15-1 Kitasato, Minami-ku, Sagamihara, Kanagawa, 252-0373, Japan

*Corresponding author: miho@fms.saitama-u.ac.jp

Contents

S1 Amino acid sequence of NLS based GFP

S2 General fluorescence microscopic observation to compare two types of molecular sensors localizations cells

S3 Identification of different cell states upon NLS based molecular sensor uptake by fluorescence microscopic observation in detail

Supplementary Figure S1

Amino acid sequences for molecular sensor of separase including NLS marked with red for NLS, yellow for adjusted linker, blue for separase recognition sequence, green for dye attached cysteine. In case of WNLS, GFP with simply removed GPKKKRKV. In case of amino acid sequences for molecular sensor of caspase-3, recognition sequence of caspase-3, DEVD and original linker sequences were denoted with blue and pink respectively. Amino acid sequences for molecular

sensor of caspase-9 is comparably marked with blue for recognition sequence (LEHD) and yellow for referred linker sequences.

MASMTGGQQMGR **GPKKKRKV** MSKGEELFTG VVPILVELDG DVNGHKFSVS GEGEGDATYG KLTLKFISTT GKLPVPWPTL VTTLTYGVQC FSRYPDHMKR HDFFKSAMPE GYVQERTISF KDDGNYKTRA EVKFEGDTLV NRIELKGIDF KEDGNILGHK LEYNYNSHNV YTTADKQKNG IKANFKTRHN IEDGSVQLAD HYQQNTPIGD GPVLLPDNHY LSTQSALLKD PNEKRDHMVL LEFVTAAGSGSSG **DREIMRE**GTC ELYK GG HHHHHH

MASMTGGQQMGR MSKGEELFTG VVPILVELDG DVNGHKFSVS GEGEGDATYG KLTLKFISTT GKLPVPWPTL VTTLTYGVQC FSRYPDHMKR HDFFKSAMPE GYVQERTISF KDDGNYKTRA EVKFEGDTLV NRIELKGIDF KEDGNILGHK LEYNYNSHNV YTTADKQKNG IKANFKTRHN IEDGSVQLAD HYQQNTPIGD GPVLLPDNHY LSTQSALLKD PNEKRDHMVL LEFVTAAGSGIT **DEVD**GTC ELYK GG HHHHHH

MASMTGGQQMGR MSKGEELFTG VVPILVELDG DVNGHKFSVS GEGEGDATYG KLTLKFISTT GKLPVPWPTL VTTLTYGVQC FSRYPDHMKR HDFFKSAMPE GYVQERTISF KDDGNYKTRA EVKFEGDTLV NRIELKGIDF KEDGNILGHK LEYNYNSHNV YTTADKQKNG IKANFKTRHN IEDGSVQLAD HYQQNTPIGD GPVLLPDNHY LSTQSALLKD PNEKRDHMVL LEFVTAAGSGSSGIT **LEHD**GTC ELYK GG HHHHHH

Supplementary Figure S2

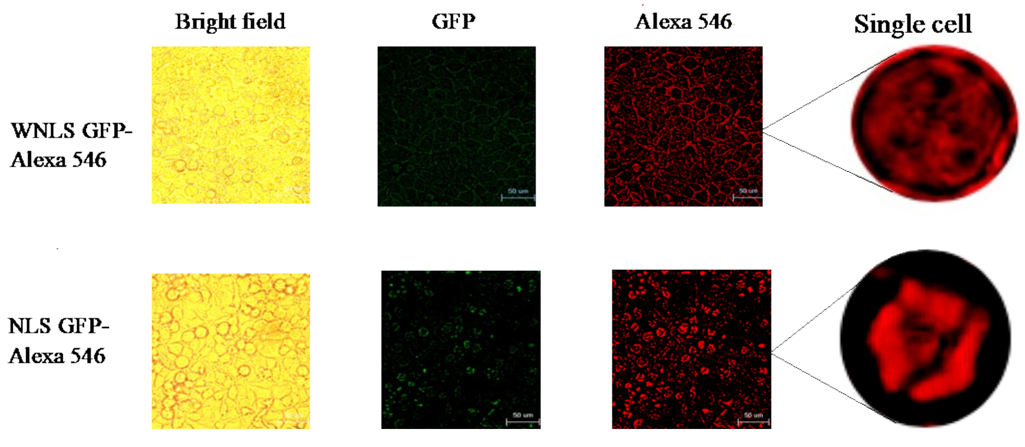

Figure S2: General fluorescence microscopic observation to compare two types of molecular sensor localizations in cells. It appeared that not localized type of molecular sensor (WNLS based) stays at endosome surrounding the nucleus to disperse into the cytosol. On the other hand, nucleus localized type of molecular sensor (NLS based) appeared to be accumulated inside nucleus.

Supplementary Figure S3

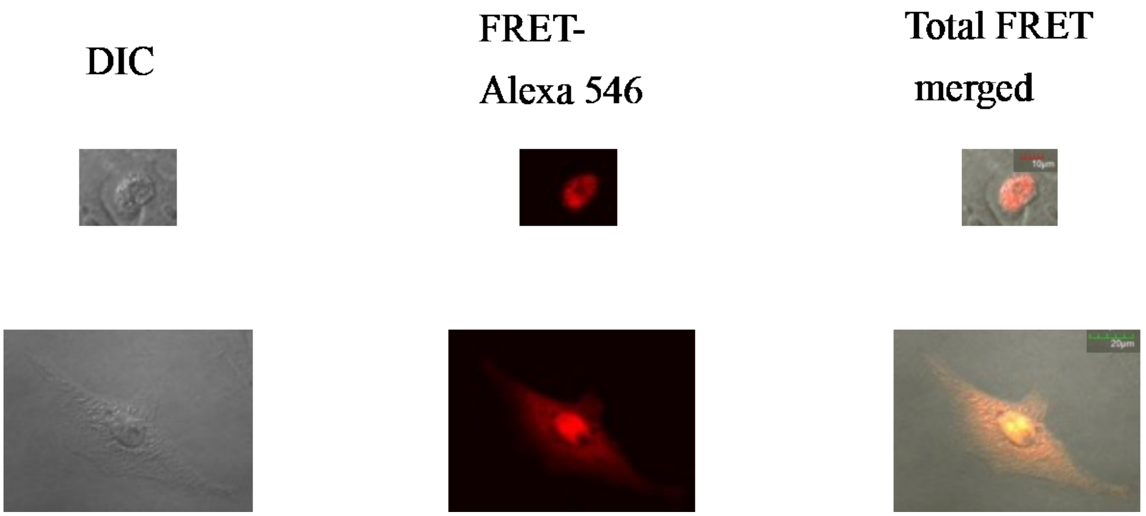

Figure S3: Identification of different cell states upon NLS based molecular sensor uptake using fluorescence microscopic observations in detail. Although forms of introduced cells were varied as a spherical shape or spread one, they still exhibited identical localization patterns for molecular sensors.

Supplementary Table S1

Estimated Förster Radius

Probe type	J(l) (M-1cm3)	n^{-4}	eA(MAX)	QYD	R_0 (nm) [k^2 = 2/3]
GFP(caspase-3)-Alexa Fluor 532	1.27E-11	0.32	81000	0.68	10.8
GFP(caspase-3)-Alexa Fluor 546	1.03E-11	0.32	104000	0.68	10.5
GFP(caspase-3)-Alexa Fluor 555	2.05E-11	0.32	150000	0.68	11.7
GFP(caspase-3)-Alexa Fluor 594	5.63E-12	0.32	73000	0.68	9.5
GFP(caspase-3)-Alexa Fluor 633	6.45E-12	0.32	239000	0.68	9.7
GFP(caspase-3)-Alexa Fluor 647	7.83E-12	0.32	132000	0.68	10.0
GFP(caspase-3)-Alexa Fluor 660	3.65E-12	0.32	184000	0.68	8.8
GFP(caspase-3)-Alexa Fluor 750	9.89E-13	0.32	240000	0.68	7.1
